# Correcting for cell-type effects in DNA methylation studies: reference-based method outperforms latent variable approaches in empirical studies

**DOI:** 10.1186/s13059-017-1148-8

**Published:** 2017-01-30

**Authors:** Mohammad W. Hattab, Andrey A. Shabalin, Shaunna L. Clark, Min Zhao, Gaurav Kumar, Robin F. Chan, Lin Ying Xie, Rick Jansen, Laura K. M. Han, Patrik K. E. Magnusson, Gerard van Grootheest, Christina M. Hultman, Brenda W. J. H. Penninx, Karolina A. Aberg, Edwin J. C. G. van den Oord

**Affiliations:** 1 Center for Biomarker Research and Precision Medicine, Virginia Commonwealth University, Richmond, VA USA; 2 Department of Psychiatry, VU University Medical Center/GGZ inGeest, Amsterdam, The Netherlands; 3 Department of Medical Epidemiology and Biostatistics, Karolinska Institutet, SE-171 77 Stockholm, Sweden

## Abstract

Based on an extensive simulation study, McGregor and colleagues recently recommended the use of surrogate variable analysis (SVA) to control for the confounding effects of cell-type heterogeneity in DNA methylation association studies in scenarios where no cell-type proportions are available. As their recommendation was mainly based on simulated data, we sought to replicate findings in two large-scale empirical studies. In our empirical data, SVA did not fully correct for cell-type effects, its performance was somewhat unstable, and it carried a risk of missing true signals caused by removing variation that might be linked to actual disease processes. By contrast, a reference-based correction method performed well and did not show these limitations. A disadvantage of this approach is that if reference methylomes are not (publicly) available, they will need to be generated once for a small set of samples. However, given the notable risk we observed for cell-type confounding, we argue that, to avoid introducing false-positive findings into the literature, it could be well worth making this investment.

Please see related Correspondence article: https://genomebiology.biomedcentral.com/articles/10/1186/s13059-017-1149-7 and related Research article: https://genomebiology.biomedcentral.com/articles/10.1186/s13059-016-0935-y

## Correspondence

Tissues often consist of multiple cell types that show different methylation patterns. In association studies, these differences can cause spurious findings when the relative abundance of the cell types is related to the outcome of interest. The inclusion of cell-type proportions as covariates will prevent such false positives. To avoid performing cell counts on all subjects in the study, these proportions can be estimated by using a small set of reference methylomes obtained using DNA from sorted cells [1]. However, reference methylomes might not always be (publicly) available and or be difficult to generate. In these scenarios, latent variables obtained by a decomposition of the methylation data can be used as a proxy for cell-type proportions. McGregor et al. [2] performed an extensive simulation study comparing one reference-based and seven latent variable methods. Although not always the best method, the reference-based method performed well. For scenarios where no reference is available, the authors recommended the use of surrogate variable analysis (SVA) [3], which performed adequately in all simulation scenarios.

As the recommendation by McGregor and colleagues [2] was based mainly on simulated data, we studied SVA in two large-scale empirical studies. The first involved 1149 Dutch subjects (825 cases with depression and 324 controls) aged 18–65 years [4] and the second 1448 Swedish subjects (774 schizophrenia cases and 674 controls) aged 25–92 years [5, 6]. Using whole-blood samples from six US subjects, cell populations were isolated by positive selection using EasySep™ kits (Stemcell Technologies), which apply magnetic nanoparticles coated with antibodies against a particular surface antigen (CD molecules). Specifically, we used CD3, CD19, CD20, CD14, and CD15 to isolate all common cell types in blood. All methylation data were generated using methyl-CG binding domain sequencing (MBD-seq) [7, 8], but the schizophrenia study was conducted on an older sequencing platform with a slightly different laboratory protocol. We used a permutation test to examine whether our top methylome-wide association study (MWAS) results were enriched for sites showing significant methylation differences among cell types. The MBD-seq procedure assays almost all 28 million common CpGs in the human genome. As the SVA package could not process all sites simultaneously, it was performed on 12 randomly selected subsets of 100,000 CpG sites.

Table 1 indicates that, if no cell-type correction is applied, MWAS findings show a greater than sixfold enrichment of CpG sites exhibiting cell-type differences in methylation. This was consistent with the significant case-control differences in estimated cell-type proportions (across cell types/studies, the median *P* value was 8.0 × 10^–5^) and stresses the need to control for this confounder. The enrichment disappears when using the reference-based method. By contrast, significant enrichment remained after SVA correction in all studied scenarios. The performance of SVA was associated with the number of surrogate variables (SVs), which varied considerably across the 12 randomly selected CpG subsets within each study. However, even when as many as 84 SVs were included, SVA failed to control for more-subtle cell-type effects. To enable a simultaneous analysis of all sites, analyses were repeated using principal component analysis (PCA) [9], which also corrects for cell types by using latent variables. However, this did not improve results.

**Table 1:** Comparison of reference-based and latent variable cell-type corrections in two empirical DNA methylation studies

	Depression MWAS study				Schizophrenia MWAS study			
	Enrich. ratio	Enrich. *P* value	Number of SVs	Increase *r* ^2^	Enrich. ratio	Enrich. *P* value	Number of SVs	Increase *r* ^2^
No cell-type correction	6.04	<0.001	–	–	6.13	<0.001	–	–
Reference-based correction	1.08	0.084	–	0.0%	1.02	0.029	–	0.0%
SVA subset 1	1.11	0.001	84	8.1%	6.54	0.001	5	0.8%
SVA subset 2	1.26	0.001	83	8.7%	7.24	<0.001	10	3.3%
SVA subset 3	1.85	0.004	19	2.0%	6.45	0.001	5	0.9%
SVA subset 4	1.28	<0.001	83	9.3%	7.01	0.001	12	2.8%
SVA subset 5	3.05	0.001	14	1.9%	6.79	0.002	6	1.2%
SVA subset 6	1.30	0.001	81	7.8%	6.59	<0.001	6	0.9%
SVA subset 7	1.07	<0.001	78	9.2%	6.42	<0.001	4	0.7%
SVA subset 8	1.28	0.004	79	9.2%	6.48	<0.001	4	0.7%
SVA subset 9	1.13	0.003	84	9.2%	7.46	0.001	10	2.7%
SVA subset 10	1.07	0.003	80	8.4%	6.71	0.003	8	1.7%
SVA subset 11	1.06	0.006	21	2.7%	6.48	<0.001	8	1.3%
SVA subset 12	1.17	0.004	84	7.4%	6.49	0.001	5	0.8%

We used a permutation test to examine whether our top methylome-wide association study (MWAS) results were enriched for sites showing significant methylation differences among cell types. Our test preserved the correlation structure of the data by shifting the CpG coordinates of the case-control and cell-type MWAS by a single random number in each permutation. We examined multiple cut-offs (1, 5, and 10%) to define “top results” in the case-control and cell-type data and selected the most significant combination. We accounted for this “multiple testing” by also selecting the most significant finding in each permutation. The “No cell-type correction” model includes laboratory technical covariates, age, and sex. The other models include these same covariates where the “Reference-based correction” model adds estimates of cell-type proportions, and the SVA models add latent variables. “Enrich. ratio” is the ratio of the number of CpGs showing methylation differences between cell types among the top MWAS finding relative to the number expected under the null hypotheses assuming no enrichment; “Enrich. *P* value” is the probability under this null hypothesis, as determined through permutations; “Number of SVs” is the number of latent variables selected by SVA; “Increase *r* ^2^” is additional variance explained by SVs in case-control status compared with a multiple regression model that included technical covariates, age/sex, and cell-type estimates. *SV* surrogate variable, *SVA* surrogate variable analysis

The use of a reference-based method ensures that only variation linked to differences in cell-type proportions is eliminated. SVA can eliminate any general source of variation in the methylation data. This carries the risk of missing true signals when some SVs capture part of the disease processes (e.g., a pathway). Table 1 reports additional variance explained by SVs in case-control status compared with a multiple-regression model that included technical covariates, age/sex, and cell-type proportions. Depending on the number of SVs, the additional variance ranged from 1 to 9%. This illustrates the risk of SVA potentially eliminating true signals in a MWAS. To mitigate this risk, one could avoid regressing out SVs associated with the case-control status. However, as cell-type proportions are related to both case-control status and SVs, such a modified analysis might be even less effective in controlling for cell-type effects.

With empirical data, SVA did not adequately correct for cell-type effects, had somewhat unstable performance, and carried a risk of missing true disease signals. The PCA suggested that these limitations might not be specific to SVA but are inherent to the use of latent variables—that is, whereas these corrections assume that cell-type heterogeneity impacts many sites, cell-type effects seem more subtle and cannot be fully captured by just the main latent variables. For this reason, we expect our findings to generalize to methylation platforms other than MBD-seq. By contrast, the reference-based method was superior in all respects. If reference methylomes are not (publicly) available for a given tissue and methylation assay, they will need to be generated once for a small set of samples. However, given the notable risk we observed for cell-type confounding, to avoid introducing false-positive findings into the literature it could be well worth making this investment.

## Abbreviations

MBD-seq: Methyl-CG binding domain sequencing
MWAS: Methylome-wide association study
PCA: Principal component analysis
SV: Surrogate variable
SVA: Surrogate variable analysis
